# Stem Cell Therapies in Retinal Disorders

**DOI:** 10.3390/cells6010004

**Published:** 2017-02-02

**Authors:** Aakriti Garg, Jin Yang, Winston Lee, Stephen H. Tsang

**Affiliations:** 1 Jonas Children’s Vision Care, and Bernard & Shirlee Brown Glaucoma Laboratory, Department of Ophthalmology, Columbia University Medical Center, Edward S. Harkness Eye Institute, 635 West 165th Street, Box 112, New York, NY 10032, USA; ag2965@columbia.edu (A.G.); yangjinchina324@gmail.com (J.Y.); wl2355@cumc.columbia.edu (W.L.); 2 Department of Pathology & Cell Biology, Columbia University College of Physicians and Surgeons, New York, NY 10032, USA

**Keywords:** stem cells, gene therapy, retina, disease modeling

## Abstract

Stem cell therapy has long been considered a promising mode of treatment for retinal conditions. While human embryonic stem cells (ESCs) have provided the precedent for regenerative medicine, the development of induced pluripotent stem cells (iPSCs) revolutionized this field. iPSCs allow for the development of many types of retinal cells, including those of the retinal pigment epithelium, photoreceptors, and ganglion cells, and can model polygenic diseases such as age-related macular degeneration. Cellular programming and reprogramming technology is especially useful in retinal diseases, as it allows for the study of living cells that have genetic variants that are specific to patients’ diseases. Since iPSCs are a self-renewing resource, scientists can experiment with an unlimited number of pluripotent cells to perfect the process of targeted differentiation, transplantation, and more, for personalized medicine. Challenges in the use of stem cells are present from the scientific, ethical, and political realms. These include transplant complications leading to anatomically incorrect placement, concern for tumorigenesis, and incomplete targeting of differentiation leading to contamination by different types of cells. Despite these limitations, human ESCs and iPSCs specific to individual patients can revolutionize the study of retinal disease and may be effective therapies for conditions currently considered incurable.

## 1. Introduction

### Stem Cell Transplantation

Stem cell therapy has long been considered a promising mode of treatment for retinal conditions. Human embryonic stem cells (hESCs) were once considered the only promising source of replacement cells in regenerative medicine. However, hESCs are associated with numerous drawbacks, including the concomitant administration of lifelong immunosuppressive therapy and limited effectiveness. Thus, when patient-specific induced pluripotent stem cell (iPSC) therapy was developed, exploration of disease pathophysiology, novel drug development, and the possibility of stem cell therapy in retinal disorders were forever changed [1].

The retina, particularly the subretinal space, is advantageous to stem cell transplantation as the eye is relatively immune privileged. The blood–ocular barrier protects the subretinal space by antigen-specific inhibition of responses of cellular and humoral immune systems, provided that it is not physically compromised during transplantation or due to the underlying disease pathology [2]. In such cases, the immunogenicity remains a challenge in hESC-derived transplantation, but can be mitigated by the iPSC approach. The propensity for tumorigenesis—such as the formation of teratomas, as is often the case with iPSCs, which are prone to epigenetic and transcriptional aberrations [3,4]—is a recurring complication, secondary to transplantation, that can be treated with plaque brachytherapy or *proton beam* radiotherapy without high risk of life-threatening consequences. Additionally, the eye is easily accessible for monitoring by exam and with several high-resolution imaging modalities, without the need for tissue biopsies pre- and post-transplantation.

## 2. Embryonic Stem Cells (ESCs)

A landmark study of ESCs by Schwartz et al. successfully transplanted 5 × 10^4^ human ESC-derived retinal pigment epithelial (RPE) cells into one eye of two patients with two different forms of macular degeneration, dry age-related macular degeneration (AMD) and advanced Stargardt macular dystrophy. The initial report found that human ESC-derived RPE cells showed no signs of rejection, ectopic tissue formation, tumorigenicity, or hyperproliferation 4 months after transplantation [5]. Further preliminary results from phase 1/2 in 18 patients (9 Stargardt; 9 AMD) confirmed long-term safety and graft survival with adverse events in several patients limited to the surgical procedure or immunosuppressive regimen [6]. The authors reported increases in various functional endpoints, such as best-corrected visual acuity and quality-of-life measures, but acknowledged the need for more rigorous structure–functional relationships with clinical tests such as microperimetry, autofluorescence imaging, and optical coherence tomography scanning [6,7].

Methods for generating a master cell bank of human embryonic cell stems are detailed as follows from the previously mentioned study by Schwartz et al. [5]. This study used human ESC line Ma09 cells, which is classified as an allotransplantation. In the Schwartz et al. trial, human ESC differentiation resulted in greater than 99% pure RPE, with markers of pluripotency such as octamer-binding transcription factor 4 (OCT4), NANOG, and sex-determining region Y-box 2 (SOX2) substantially downregulated, and paired box 6 (PAX6) and RPE markers (RPE65, bestrophin 1 (BEST1), and microphthalmia-associated transcription factor (MITF)) significantly more highly expressed [5].

## 3. Induced Pluripotent Stem Cells (iPSCs)

The development of iPSCs allowed for a source of retinal cells for transplantation, much more cost-effective methods of drug testing, and the development of models that, at times, mimic human disease better than animal models, which do not always have physiology that is comparable to humans.

In 2007, Takahashi et al. published a method describing the creation of iPSCs, skin fibroblasts were first transduced with viral constructs expressing four transcription factors—OCT4, SOX2, Krüppel-like factor 4 (KLF4), and C-MYC [8,9,10,11]—that allowed mature cells to return to a pluripotent state similar to that seen in ESCs [10].

## 4. Success of iPSCs

Following the paradigm of all translational research, preclinical efficacy of iPSCs must be proven prior to use in human trials. Studies of RPE-based disorders have been shown to be the best candidates for iPSC modeling, given accessibility through manual dissection and expansion on an assortment of substrates, behavior that mimics primary human prenatal in vitro, as well as ease of monitoring of maturation state through distinct morphological features [12,13].

Novel treatment approaches have also been promising. The methods of a recent study by Li et al. on the transplantation of iPSC-derived RPE cells are as follows. Skin biopsy had been performed to obtain fibroblasts, which were subsequently cocultured with mitomycin-C-treated PA6 feeder cells, which possess stromal-derived inducing activity (SIDA) and promote RPE differentiation. As described by Takahashi et al., vectors carrying transcription factors OCT4, SOX2, KLF4, and MYC were used to reprogram cells [10,14]. Morphology and function of iPSCs was characterized by immunohistochemistry, electron microscopy, and functional analysis. iPSC-derived RPE cells were grafted subretinally into the subretinal space of mouse eyes. This study found that human iPSC-derived RPE cells were successful in restoring retinal function, as assessed by electroretinography in a mouse possessing the mutation in a gene known to be responsible for certain types of retinitis pigmentosa (RP) [14].

Maeda et al.’s group found that in comparison to isolated wild-type mouse primary RPE (mpRPE) cells, iPSC-RPE cells maintained expression of certain visual cycle proteins during cell culture, while mpRPE cells rapidly lost this trait. Specifically, iPSC-RPE cells produced the visual chromophore, 11-*cis*-retinal, and formed retinosomes in vitro. Further, visual function was recovered when iPSC-RPE cells were transplanted into both blind *Lrat^−/−^* and *Rpe65^−/−^* mice. Additionally, iPSC-RPE cells were found to replace dysfunctional RPE cells on histological analysis. Thus, a functional visual cycle was exhibited in vitro and in vivo by iPSC-RPE cells [15]. Further, it is no surprise that studies of retinitis pigmentosa in animal models using iPSC transplants have shown success in several studies. Additionally, improved visual-guided behaviors were noted for 6 weeks after transplantation in another preclinical RP model that received iPSC-derived photoreceptor transplants [16].

## 5. Stem Cells and Retinal Conditions

Retinal degenerative diseases such as age-related macular degeneration, Stargardt disease, and retinitis pigmentosa, are phenotypically diverse but have shown significant promise in being treated with iPSCs. Age-related macular degeneration, the leading cause of blindness worldwide in those above age 55 years, currently affects 1.75 million people in the USA and will affect nearly 196 million people worldwide by 2020 [17]. Stargardt disease is the most prevalent inherited macular dystrophy and is the leading cause of juvenile macular degeneration, with a prevalence of 1 in 8000–10,000 [18]. The term retinitis pigmentosa encompasses a heterogenous group of progressive retinal degenerative disorders with a worldwide prevalence of 1 in 3500–5000 individuals [19]. These and many other currently untreatable retinal conditions are the subject of many clinical trials using iPSCs to perform RPE transplantation [20].

The role of iPSCs in treatment of retinal conditions lies in their success in creating the makeup of the retina. Many types of retinal cells, including those of the RPE, photoreceptors, and ganglion cells, have been differentiated from iPSCs [21,22]. RPE cells are monolayers with pigment, making them easier to purify and isolate than other types of cells [23]; they were thus the first types of retinal cells to be differentiated from iPSCs.

Preclinical testing became possible with the development of iPSC cell lines from monkeys and subsequent differentiation into RPE cells [24]. The genetic similarities between humans and nonhuman primates allowed for the testing of the safety and efficacy of iPSC cells in vivo, using techniques that are to be employed in clinical trials. Additionally, the possibility of immune rejection was examined. Flow cytometry-based assays have shown that phagocytosis of iPSC-derived RPE is comparable in function to those found in RPE cell lines ARPE-19 and human fetal RPE (hfRPE] [25]. Future directions in retinal cell development include the growth of iPSC-derived photoreceptors and ganglion cells.

iPSCs play a significant role in the development of animal models of conditions that involve not only one gene, but many. These polygenic diseases include age-related macular degeneration, which is the leading case of irreversible blindness in the world [26].

## 6. Modeling Retinal Diseases in a Dish

Disease modeling in a dish provides numerous advantages over traditional human disease research, ranging from epidemiology to animal model experiments. Obstacles inherent to these conventional methods include limited availability of human tissue samples, inability to immortalize and manipulate cells while still maintaining their true physiologic properties, and variation between human diseases and their animal models [1]. The study of retinal disease poses unique challenges that arguably makes these conditions even more difficult to study. In retinal disease, like neurological disease, cell purification and cell development are difficult to study because affected tissue is not readily available. This makes cellular programming and reprogramming technology (CPART) especially useful, as modeling diseases in this manner allows for the study of living cells that have genetic variants and background that are specific to patients’ specific diseases [27].

Current retinal differentiation protocols for iPSCs can be divided into two categories, default differentiation and directed differentiation. In the first, cells are cultured in the absence of extrinsic growth factors, while in the second, differentiation is dependent on the addition of extrinsic transcription factors, small molecules, and proteins. Choice of protocol is dependent on purification goals and research aims, as no differentiation protocol provides complete efficiency in the production of retinal cells [28].

The underlying assumption in modeling diseases in vitro is that cellular deficiencies are measurable in this state and can recreate the phenotype that is demonstrated in human diseases. The concern here lies in the fact that many diseases are too variable in phenotype to be recreated in a meaningful way [27]. Such variation is particularly evident in iPSC lines.

Mechanisms of variation include variable expression profiles, female line X-inactivation, genetic instability, partial reprogramming, retention of epigenetic marks, and differentiation potential [29]. Teratoma formation is considered the gold standard criterion for the validation of pluripotency, but is not without flaws. It is considered difficult to standardize, primarily qualitative, and has questionable value in the modeling of in vitro disease [30].

Efforts to decrease variation naturally include increasing sample size. This would include creating disease team consortiums and increasing the number of analyzed patients and controls. Additionally, creating cohorts of patients who respond similarly to drugs and present with similar forms of diseases would allow for more specialized study [27]. The fact that iPSCs are a self-renewing resource will allow for the accumulation of this large sample size; scientists can experiment with an unlimited number of pluripotent cells to perfect the process of targeted differentiation, transplantation, and more, for personalized medicine [1].

A recent study by Yang et al. has showed a role for iPSCs in the determination of pathophysiology in age-related macular degeneration, which is characterized by the loss of RPE cells. iPSCs derived from the cells of patients with age-related macular degeneration allowed these scientists to analyze the downregulatory action of the age-related maculopathy susceptibility 2/high-temperature requirement A serine peptidase [*ARMS2/HTRA1*) risk alleles on superoxide dismutase 2 (SOD2) defense, which they found may be ultimately responsible for oxidative damage to the RPE [31].

## 7. Limitations of Stem Cell Use

Research with hESC has proven to be challenging, both from a scientific, ethical, and political perspective.

There are also several complications of iPSC transplants. Integration requires that grafted photoreceptors assume the correct orientation, in which an inner synapse and an outer photoreceptive segment are positioned against host inner retina and RPE, respectively. Several groups have demonstrated successfully overcoming this obstacle. Eiraku et al. developed a three-dimensional multilayered autonomous optic cup containing both rods and cones that spontaneously curves in an apically convex configuration [32,33]. Additionally, Zhong et al. created retinal cups containing all major cell types in proper configuration and were able to demonstrate the beginning of outer-segment disc formation and photosensitivity [34]. Further, Assawachananont et al. were able to successfully demonstrate retinal sheet transplantation therapy by grafting outer nuclear layer in an advanced retinal degeneration model [35].

Other complications include gliosis hampering transplantation into the retina as well as epigenetic profile, DNA sequence, and copy number heterogeneity between cell lines produced at different laboratories [36]. The latter may be ameliorated by utilizing a combination of reprogramming transcription factors or adjusting DNA methylation status that allow for the quality and heterogeneity of iPSC lines [37].

Of significant concern in iPSC use is tumorigenesis due to the persistence of undifferentiated iPSCs at the end of reprogramming and differentiation protocol. The extension of this procedure has helped to address these concerns, as none of the mice received transplants under the new protocol developed tumors throughout their lifetimes [14]. Additionally, the chances of tumorigenesis has been greatly reduced with the advent of novel gene transfer methods, which include membrane-permeable peptides [38,39] and episomal plasmids [40,41], which are not integrated into the genome [42]. These advancements corroborate the safety of the transplantation of iPSC grafts.

Lastly, the pluripotency of iPSCs is their greatest strength, but can also be viewed as their principal disadvantage, as isolating and identifying desired cell types is challenging. If targeted differentiation is not achieved, experiments can be confounded by unwanted cell types that have contaminated the same dish [43]. As mentioned in the next section, no current differentiation protocol has achieved complete fidelity [28].

## 8. Conclusions

Human ESCs and iPSCs specific to individual patients are a beneficial tool for the study of retinal disease and may be effective therapies for conditions currently considered incurable. Minimizing the chances of tumorigenicity and precisely targeting differentiation are the most significant challenges that must be overcome in order to make iPSC therapy a reality to treat RPE-related disorders. Ultimately, being able to model retinal conditions in vitro allows for the study of the cell development that is truly personalized to patients’ disease-specific genetic variants.
